# Evaluating the Quality of Cardiovascular Disease Information From AI Chatbots: A Comparative Study

**DOI:** 10.7759/cureus.88085

**Published:** 2025-07-16

**Authors:** Joshua Singavarapu, Amber Khemlani, Menachem Jacobs, Eli Berglas, Jason Lazar, Abdo Kabarriti

**Affiliations:** 1 Cardiology, State University of New York Downstate Health Sciences University, Brooklyn, USA; 2 Urology, State University of New York Downstate Health Sciences University, Brooklyn, USA

**Keywords:** arrhythmia, artificial intelligence, cardiology, chatbot, chatgpt, claude, gemini, heart failure, myocardial infarction, perplexity

## Abstract

Artificial intelligence (AI) is increasingly being utilized as an informational resource, with chatbots attracting users for their ability to generate instantaneous responses. This study evaluates the understandability, actionability, readability, quality, and misinformation in medical information provided by four prominent chatbots - Bard, ChatGPT 3.5, Claude 2.0, and Perplexity - on three prevalent cardiovascular diseases (CVDs): myocardial infarctions, heart failure, and arrhythmias. These chatbots were used because of their popularity and high usage rates among chatbots. Using Google Trends, the top five U.S. search queries related to heart attack, arrhythmia, and heart failure from September 29, 2018, to September 29, 2023, were identified. The top five queries were chosen in relation to these topics because they accounted for over 80% of the public's searches related to these topics. The chatbot responses were blinded and analyzed by two evaluators using DISCERN for quality, Patient Education Materials Assessment Tool (PEMAT) for understandability and actionability, and Flesch-Kincaid scores for readability. Statistical tests included the Kruskal-Wallis test for DISCERN, the chi-square test for PEMAT, and one-way ANOVA for Flesch-Kincaid scores. Bard generated responses with a statistically lower Flesch-Kincaid reading score than the other chatbots. Bard and ChatGPT 3.5 provided more actionable responses. Among the CVD topics, “heart attack” yielded lower-grade-level responses and more actionable information compared to “arrhythmia” and “heart failure.” This study is among the first to assess AI credibility in disseminating cardiovascular information. It highlights how acute pathologic events may prompt more actionable and accessible chatbot responses. As AI continues to evolve, collaboration among healthcare professionals, researchers, and developers is crucial to ensuring the safe and effective use of AI in patient education and public health.

## Introduction

Natural language processing (NLP) and large language model (LLM) technologies have the potential to improve patient access to medical information [1]. Pre-trained LLMs, such as ChatGPT and other artificial intelligence (AI) chatbots, are advanced NLP tools capable of interacting with and generating human-like text. Unlike traditional machine learning models, LLMs undergo self-supervised processing of large volumes of raw data, fine-tuning specific, annotated datasets to cater to end-user-specified tasks [2]. 

The shift toward digital information, underscored by the widespread use of Internet resources and search engines, has long changed how patients seek medical guidance [3]. Increasingly, individuals are starting to consider AI chatbots over traditional search methods, attracted by personalized interactions and the ability to obtain tailored, instantaneous results [4]. This has been shown by the increase in chatbot usage over the years and the mainstay of AI within information distribution. Chatbots such as Bard (now Gemini), ChatGPT 3.5, Claude 2.0, and Perplexity are being used as information sources, steadily replacing traditional search engines by interpreting and condensing large amounts of information [5]. These chatbots were chosen for this study because of their prevalence with daily users and also the ability of the selected chatbots to incorporate search engines to describe and evaluate information for the user. While this technology is still in its infancy, it holds promise in addressing the growing demand for health services, including patient education and triage. By facilitating more efficient and frequent patient interactions, AI chatbots have the potential to transform healthcare delivery by offering personalized education, symptom tracking, medication management, and lifestyle guidance for chronic disease patients [6]. This could significantly enhance healthcare access and doctor-patient communication [7]. On the other hand, there is also a limitation in AI and the information that it can provide. It has the potential for misinformation and can also be potentially mismanaged by a patient to lead them towards incorrect next steps. 

Thus, this paper will examine how Bard, ChatGPT 3.5, Claude 2.0, and Perplexity interpret prompts regarding three prevalent cardiac disorders: myocardial infarction (heart attack), arrhythmia, and heart failure. These pathologies were chosen based on the high volume of patients who experience these cardiovascular diseases (CVDs). As of 2021, there were close to 805,000 new and recurrent cases of myocardial infarctions [8]. Arrhythmias affect 1.5-5% of the population, equating to about five million Americans [9], with this number being inconclusive based on cases without symptomatic effects. In addition, heart failure cases affect, on average, about 6.7 million Americans over the age of 20, and this number is expected to rise to over 8 million cases by 2030 [10]. Given the prevalence of these CVDs and the increasing use of chatbots for medical information, the present study will evaluate responses from chatbots regarding these pathologies to assess their overall information quality, understandability, and actionability. The DISCERN scale will be used to evaluate quality, and the Patient Education Materials Assessment Tool (PEMAT) scoring system evaluates both understandability and actionability. Both of these scoring systems are publicly available and have been used over the years to evaluate medical modes of information. In this study, the use of DISCERN and PEMAT will allow a better understanding of the role chatbots have in CVD prevention, diagnosis, and treatment. 

## Materials and methods

This cross-sectional study was exempt from review and informed consent in accordance with the Common Rule, given its use of publicly available data. The top five Google (Alphabet, Inc.) search queries related to heart attack, arrhythmia, and heart failure in the US for the five years from September 29, 2018, to September 29, 2023, were identified using Google Trends. This publicly available web tool provides data on search-term volume for the Google search engine over time. The top five search queries related to each of these pathologies were identified and input into four AI chatbots: Bard, ChatGPT 3.5, Claude 2.0, and Perplexity. The top five search queries, though they seem small, were used because they covered over 80% of the general public's questions on each cardiovascular topic.

These chatbots were used in their default settings using the most updated publicly accessible version as of October 10, 2023. For each new search query, the previous conversation was deleted, and a new conversation was initiated to prevent the memory of earlier queries from influencing subsequent responses. The inputs into AI chatbots matched the exact phrasing from Google Trends. The Google Trends keywords are included in Table 1. Each response was evaluated for the quality of information using DISCERN (overall scores of 1 (low) to 5 (high) for quality of information) and the understandability and actionability domains of the PEMAT5 (scores of 0-100%, with higher scores indicating a higher level of understandability and actionability). DISCERN is an instrument that has been created for the evaluation of response quality, with the hope of creating evidence-based consumer health education [11]. PEMAT-understandability and actionability grading scales were similarly created to evaluate print materials and assess the ability of information consumers to understand and act upon what they are reading [12]. Both instruments have shown good interrater reliability. In addition, understandability was objectively evaluated by the Flesch-Kincaid reading score, which calculates grade-level difficulty based on sentence length and word length [13]. For example, a score of 8 would represent that the excerpt can be understood by someone with an eighth-grade reading ability. Each evaluation instrument is publicly available and does not require any prior permission for its use. 

**Table 1:** Top five searched key phrases for cardiovascular Google trends during 9/29/2018-9/29/2023

Google Trends keywords	Heart attack	Heart failure	Arrhythmia
Top searched key phrase	Heart attack symptoms	Congestive heart failure	Heart arrhythmia
	Heart attack signs	Heart failure symptoms	Sinus arrhythmia
	Signs of heart attack	What is heart failure	Cardiac arrhythmia
	Heart attack pain	ICD heart failure	What is arrhythmia
	Women heart attack	ICD10 heart failure	Arrhythmia symptoms

Accuracy was analyzed through the review of chatbot responses alongside the American Heart Association (AHA) society guidelines. Members of the team (A.K., J.S.) were blinded to the AI chatbot type and the other member’s assessment through a randomized list of responses made by another member of the team (M.J.). Both A.K. and J.S. proceeded to independently score the chatbot responses from November 6, 2023, to November 11, 2023. Similar methods were utilized previously to assess chatbot responses on the five most prevalent cancers [14]. 

Statistical methods

Interrater reliability with 95% confidence interval (CI) of DISCERN and PEMAT scores was assessed using weighted Cohen’s kappa and Cohen’s kappa, respectively. An average of the two ratings (one from each rater) for each DISCERN question was calculated. Average scores were stratified by chatbot, disregarding pathology (heart attack, arrhythmia, and heart failure). Kruskal-Wallis tests with post-hoc pairwise comparisons using Bonferroni correction (p-values presented were multiplied by the number of comparisons) were used to determine if there were significant differences in mean ranks across the four chatbots. Identical tests were done within each of the three pathologies to determine disease-specific chatbot differences. Lastly, to assess for differences across pathologies, the Kruskal-Wallis test with post-hoc Bonferroni correction was used to compare mean ranks of all chatbot responses within a pathology to other pathologies. Median and interquartile range (IQR) of compared DISCERN scores were reported within the texts, and average mean ranks with standard error were reported within the figures. 

Flesch-Kincaid Grade Level scores were compared using two distinct one-way ANOVAs with post-hoc Bonferroni tests. Much like DISCERN and PEMAT, comparisons were made across chatbots and across diseases. No comparisons of chatbots were computed within pathologies due to the small sample size (n = 5). All values of Flesch-Kincaid grade level were presented as mean with standard deviation (SD). To quantify the level of misinformation presented by the chatbots, the percentage of queries where the chatbot was deemed to have no misinformation (1 on a Likert scale) was recorded. This was important to incorporate in order to validate that there was no misinformation provided by the chatbots in the unlikely possibility that the response would score high on the evaluation scales. 

Cumulative PEMAT responses (N = 24 per query, per rater) were stratified into understandability (n = 17) and actionability (n = 7). For simplicity, when one grader determined a question to not be applicable to the given chatbot response, scoring from the other rater was disregarded. Three distinct chi-square tests were performed using the same grouping parameters as described for DISCERN. When indicated, post-hoc tests with Bonferroni corrections were performed. These three tests were performed independently for the understandability and actionability categories. All PEMAT-related values were reported as a percentage of cumulative questions answered “yes” by both graders, out of the total applicable questions in the category. To quantify the level of misinformation presented by the chatbots, the percentage of queries where the chatbot was deemed to have no misinformation (1 on a Likert scale) was recorded. 

All p-values were reported with any necessary Bonferroni adjustment and were considered significant when p < 0.05. To manage data and generate figures and tables, Microsoft Excel, version 16.20 (Microsoft Corporation, Redmond, WA, USA) was used. All statistical analyses were performed using IBM SPSS Statistics for Windows, Version 29.0 (released 2023, IBM Corp., Armonk, NY). 

## Results

**Figure 1:**
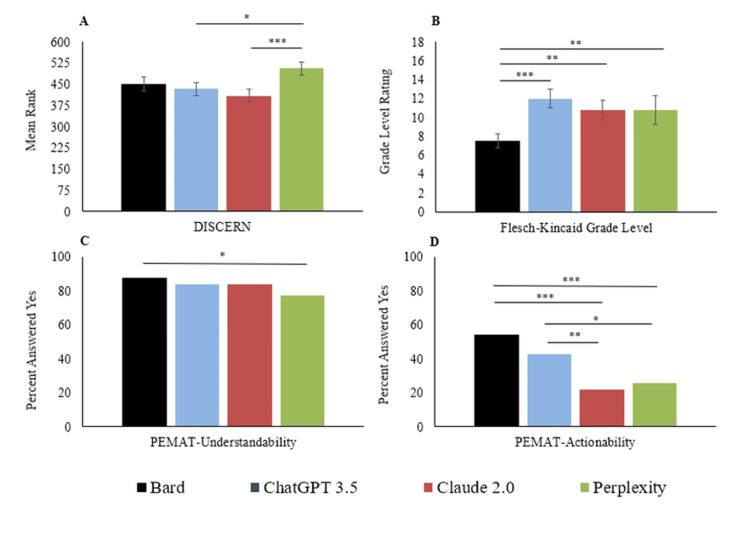
Performance of four chatbots on A) the DISCERN mean rank out of 900 total ranks, B) Flesch-Kincaid grade level (greater score = more complex), C) PEMAT-Understandability (max = 100%), and D) PEMAT-Actionability (max = 100%) Values within bars represent the median quality score on a 1 (low)-5 (high) scale. Error bars represent ± standard error. Error bars represent 95% confidence intervals. The significance level of A) Kruskal-Wallis, B. one-way ANOVA, and C-D) chi-square are depicted by * = p < 0.05, ** = p < 0.001.

**Figure 2:**
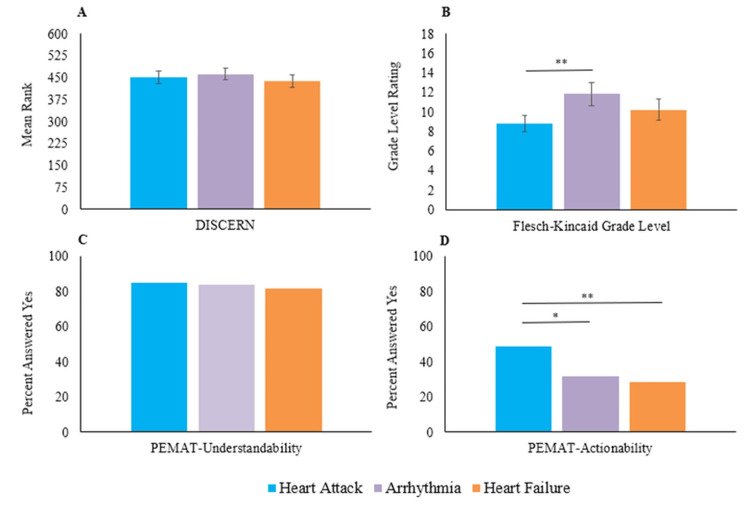
Cumulative performance of four chatbot responses to cardiovascular pathologies on A) the DISCERN mean rank out of 900 total ranks, B) Flesch-Kincaid grade level (greater score = more complex), C) PEMAT-Understandability (max = 100%), and D) PEMAT-Actionability (max = 100%) Values within bars represent median quality score on a 1 (low)-5 (high) scale. Error bars represent ± standard error. Error bars represent 95% confidence intervals. The significance level of Kruskal-Wallis, one-way ANOVA, and chi-square are depicted by * = p < 0.05 and ** = p < 0.001.

High interrater reliability was demonstrated for both the DISCERN and PEMAT scoring tools, with kappa values of 0.81 (95% CI (0.78-0.83)) and 0.79 (95% CI (0.76-0.82)), respectively (both p < 0.001), indicating strong agreement between reviewers. Across all cardiovascular queries and as seen in Figure 1, Perplexity achieved significantly higher DISCERN scores (Mdn = 4, IQR = 4) compared to ChatGPT 3.5 (Mdn = 2, IQR = 4, p = 0.011) and Claude 2.0 (Mdn = 2, IQR = 4, p < 0.001). In addition, as noted in Figure 2, no significant differences in quality were noted among chatbots for heart attack queries (p = 0.18). By contrast, within arrhythmia-related responses, Perplexity outperformed Claude 2.0 (p = 0.038), and a similar pattern was observed in heart failure queries (p = 0.022). Overall, these findings reveal distinct performance profiles among chatbots, with Perplexity demonstrating superior information quality. Table 2 also shows the ratings of chatbot responses for the three cardiovascular pathologies using DISCERN and PEMAT.

**Table 2:** Ratings of chatbot responses for three cardiovascular pathologies using the DISCERN and PEMAT scales P-values represent the result of the overall test (Kruskal-Wallis for DISCERN and chi-square for PEMAT). Pairwise comparisons are depicted as follows: (*): p < 0.05, (**): p < 0.001, (***): p < 0.0001.

		Bard	ChatGPT	Claude 2.0	Perplexity	p-value
Heart attack	DISCERN Median Score (1-5)	2.5	2	2.5	5	0.18
	PEMAT-Understandability (%)	86.46	85.56	82.22	85.56	0.86
	PEMAT-Actionability (%)	56.00	56.00	42.00	40.00	0.21
Arrhythmia	DISCERN Median Score (1-5)	3.5	2	2.5*	3.5*	0.04
	PEMAT-Understandability (%)	88.64*	84.44	86.46	73.33*	0.018
	PEMAT-Actionability (%)	58.00******	38.00*	12.00****	18.00***	< 0.0001
Heart failure	DISCERN Median Score (1-5)	3	2	1*	3.5*	0.017
	PEMAT-Understandability (%)	88.39*	81.48	83.00	72.22*	0.031
	PEMAT-Actionability (%)	48.00***	34.00	12.00**	20.00*	< 0.001

In terms of readability, Bard produced responses at a significantly lower grade level (M = 7.56, SD = 1.33) than ChatGPT 3.5 (M = 11.99, SD = 1.77, p < 0.001), Claude 2.0 (M = 10.79, SD = 1.89, p < 0.001), and Perplexity (M = 10.80, SD = 2.72, p < 0.001). Comparatively, queries about heart attacks were associated with lower reading levels (M = 8.78, SD = 1.83) than arrhythmia-related responses (M = 11.85, SD = 2.57, p < 0.001).

Understandability scores (PEMAT) varied modestly across chatbots. Bard was rated more understandable than Perplexity for arrhythmia (88.64% vs. 73.33%, p = 0.018) and heart failure responses (88.39% vs. 72.22%, p = 0.021), although no significant differences were found for heart attack queries (p = 0.86). This suggests that readability alone does not always translate into higher understandability, since the information can, at times, be unclear on where to proceed, though it uses a simpler level of vocabulary and vernacular.

Differences were more pronounced in actionability. Bard and ChatGPT 3.5 were significantly more actionable across most categories compared to Claude 2.0 and Perplexity. For arrhythmia queries, Bard achieved an actionability score of 58% compared to 12% for Claude 2.0 (p < 0.001) and 18% for Perplexity (p < 0.001). Similar trends held in heart failure responses, where Bard scored 48% versus 12% and 20% for Claude 2.0 and Perplexity, respectively. Heart attack queries, overall, yielded more actionable responses across all chatbots (48.5%) compared to arrhythmia (31.5%, p = 0.003) and heart failure (28.5%, p < 0.001).

These patterns highlight Bard’s strength in accessibility and actionability - attributes that may enhance patient engagement.

Finally, no misinformation was identified across any chatbot responses, with all queries rated as fully accurate on a five-point Likert scale. This finding suggests that current AI chatbots maintain acceptable accuracy standards when addressing common cardiovascular health queries, though this may not be true for more complex medical issues or for issues needing more actionable insights, and does not diminish the importance of professional medical consultation for individual patient care decisions. No misinformation also does not guarantee comprehensiveness or clinical adequacy. 

## Discussion

This study utilized Bard, ChatGPT 3.5, Claude 2.0, and Perplexity to interpret the top five search queries related to three prevalent CVDs: heart attack, arrhythmia, and heart failure. Previous studies have looked at how DISCERN and PEMAT can help to assess chatbot responses in oncology and urology [15]. However, in this study, the additional focus on the various parameters across cardiovascular pathologies can expand the data from a cardiovascular lens.

The DISCERN ranking shown in Figure 1A shows that Perplexity has significantly better DISCERN scores than ChatGPT 3.5 and Claude 2.0. However, this does not definitively determine that Perplexity performs overall better than the other chatbots, but rather it demonstrates a difference in quality, which is what DISCERN evaluates. To perform overall better, there is a combination of factors ranging from high performance in understandability, actionability, and readability. The Flesch-Kincaid Grade Level scores shown in Figure 1B indicate Bard has a statistically significantly lower grade level compared to the other chatbots (indicating it is easier to read). This helps with the evaluation of chatbots based on how they may educate patients, since education level cannot be assumed. These findings indicate how Bard may be a more appropriate chatbot for patients, even if Perplexity has a significantly better quality of responses.

While an easier-to-read response is important, we also evaluated understandability via the PEMAT Understandability score. Despite Bard being significantly easier to read, the understandability of its responses was only significantly higher than that of Perplexity. This finding may demonstrate an important principle: if something is easy to read, that does not necessarily mean it will be more understandable. Another critical component of the data distribution is assessing how easily the information can be utilized for a well-defined action. To understand how chatbots differed in this regard, we used the PEMAT Actionability scores. Bard and ChatGPT 3.5 had significantly higher scores than Claude 2.0 and Perplexity. The consistency in these findings is that Bard is a high-performing chatbot regarding reading ease, understandability, and actionability, but the real difference this would make for patients interested in CVDs is difficult to determine. Thus, it is challenging to assess the information that may be missing from a chatbot response. Furthermore, chatbots do not have important context, such as the patient’s past medical history and risk factors, making their responses potentially misinformed [16]. This can be demonstrated when a mild chest pain that does not warrant any further medical help can lead to a call to the emergency department by a chatbot. Therefore, chatbots are not yet ready to be at the forefront of the dissemination of medical information. However, when comparing the current state of the chatbots, the results indicate that Bard, in particular, may be well suited to give patients general information about CVDs. 

Moreover, we anticipated there may be variations in how chatbots respond across cardiovascular pathologies because of discrepancies in query prompts [17]. Thus, as seen in Figure 2D, the PEMAT Actionability across heart attack, arrhythmia, and heart failure showed that heart attack provided the responses with the highest actionability scores. It can be speculated that the acuteness of heart attack events leads chatbots to provide more aggressive recommendations, such as an emergency room visit. 

When comparing the chatbot responses of the CVD, it was also important to note that heart attack responses had significantly lower Flesch-Kincaid Reading Scores than arrhythmias (Figure 2B), which could be attributed to the complexity of terms such as arrhythmia when compared to heart attack. As “heart attack” may be a more layman term than “arrhythmia,” it is possible that chatbots have difficulty decreasing the complexity of their responses based on the complexity of the input. Future studies can investigate this concept by evaluating reading scores across more complex pathologies.

This study is limited to the use of query results from the top Google searches over the last five years, between September 29, 2018, and September 29, 2023. This Google search period also included the COVID-19 pandemic, a time when individuals had decreased access to the hospital and healthcare clinics for non-COVID-related medical emergencies, potentially altering the top Google searches during that time [18]. Another limitation of this study is that individuals have graded the chatbot responses using partially subjective scales. However, DISCERN and PEMAT have been validated [11,12] and shown to provide accurate results in regards to the quality and understandability of excerpts. As an extension, DISCERN and PEMAT Understandability scores were evaluated by medical students, which may skew the data because students may better understand the pathologies and quality of responses more so than an average individual. However, when looking at Figure 2C, PEMAT Understandability scores were similar regardless of pathology complexity. Nevertheless, this study can benefit from a wide variety of graders among different levels of learning and professions to better represent the layperson who would use this medium.

## Conclusions

Although further research is indicated to improve chatbot responses, this study highlights the potential of chatbots to improve patient education. All four chatbots are similar in some metrics, but there were key differences in actionability, grade level, and quality scores. Bard produced responses with a statistically lower grade level score compared to the three other chatbots, and Bard and ChatGPT 3.5 produced statistically significant actionable responses. Among the CVDs evaluated, it is also important to note that “heart attack” produced lower grade level responses than “arrhythmia” and more actionable responses than both “arrhythmia” and “heart failure.” To our knowledge, there have not been prior studies that evaluate chatbot responses from the cardiology patient perspective. This would be one of the first, if not the first, studies to assess AI credibility for the future of the dissemination of cardiovascular information for patients. This study interprets the significance of cardiovascular conditions when prompting chatbots, with a hypothesis developed on how acute pathologic events may call for more actionable, easier-to-read responses. However, the risks of misinformation and the variability in response quality across chatbots call for cautious integration and continuous validation of these technologies before they can be reliably deployed in routine clinical practice. Though these findings are preliminary, they can help direct attention as AI is incorporated into medicine. As AI advances, ongoing research and collaboration between healthcare professionals, researchers, and technology developers will ensure the safe and effective use of AI in empowering patients and improving public health outcomes. 
